# Potential Benefits and Challenges of Quantifying Pseudoreplication in Genomic Data with Entropy Statistics

**DOI:** 10.3390/e26090805

**Published:** 2024-09-21

**Authors:** Eric J. Ward, Robin S. Waples

**Affiliations:** 1 Northwest Fisheries Science Center, National Marine Fisheries Service, National Oceanic and Atmospheric Administration, 2725 Montlake Blvd. East, Seattle, WA 98112, USA; 2 School of Aquatic and Fishery Sciences, University of Washington, Seattle, WA 98195, USA; robinw3@uw.edu

**Keywords:** entropy, pseudoreplication, genomics

## Abstract

Generating vast arrays of genetic markers for evolutionary ecology studies has become routine and cost-effective. However, analyzing data from large numbers of loci associated with a small number of finite chromosomes introduces a challenge: loci on the same chromosome do not assort independently, leading to pseudoreplication. Previous studies have demonstrated that pseudoreplication can substantially reduce precision of genetic analyses (and make confidence intervals wider), such as *F_ST_* and linkage disequilibrium (LD) measures between pairs of loci. In LD analyses, another type of dependency (overlapping pairs of the same loci) also creates pseudoreplication. Building on previous work, we explore the potential of entropy metrics to improve the status quo, particularly total correlation (TC), to assess pseudoreplication in LD studies. Our simulations, performed on a monoecious population with a range of effective population sizes (*N_e_*) and numbers of loci, attempted to isolate the overlapping-pairs-of-loci effect by considering unlinked loci and using entropy to quantify inter-locus relationships. We hypothesized a positive correlation between TC and the number of loci (L), and a negative correlation between TC and *N_e_*. Results from our statistical models predicting TC demonstrate a strong effect of the number of loci, and muted effects of *N_e_* and other predictors, adding support to the use of entropy-based metrics as a tool for estimating the statistical information of complex genetic datasets. Our results also highlight a challenge regarding scalability; computational limitations arise as the number of loci grows, making our current approach limited to smaller datasets. Despite these challenges, this work further refines our understanding of entropy measures, and offers insights into the complex dynamics of genetic information in evolutionary ecology research.

## 1. Introduction

One consequence of the genomics revolution is that it is now relatively easy and inexpensive to generate large numbers of genetic markers—commonly 10^3^–10^7^ loci, even for non-model species. This has greatly increased statistical power for many traditional genetic analyses; when combined with detailed information about structure of the genome, it has also opened up possibilities to address qualitatively new questions in evolutionary ecology [1,2,3]. An important limitation of this wealth of new genomics data is that in real organisms, all of these loci have to be packaged into a small number of chromosomes (mean chromosome numbers are 11, 13, and 25 for invertebrates, vascular plants, and vertebrates, respectively; [4]). Because crossovers leading to recombination occur on average only a bit over once per generation within each chromosome arm [5,6], syntenic loci do not in general assort independently and hence do not provide independent information about evolutionary processes. This lack of independence creates pseudoreplication, which reduces precision of genetic analyses, compared to a hypothetical scenario in which all genetic markers are independent. Physical linkage limits precision of a wide range of common genetic indices that average results across individual loci, including measures of genetic differentiation like *F_ST_*.

A second kind of lack of independence also affects measures of linkage disequilibrium (LD), which is the non-random association of alleles at different pairs of gene loci [7]. A common measure of LD is *r*^2^ [8], which is the squared correlation coefficient between genotypes at the two loci involved. In a dataset with *L* loci, these can be stored in a triangular matrix with *L*(*L* − 1)/2 ≈ *L*^2^/2 different pairs of loci. Of these pairs, only *L*/2 are completely independent because they do not share any loci. In general, any pair of loci, *i,j*, will share one locus with 2(*L* − 2) other pairs, so the only truly independent set is [*r*_1,2_^2^, *r*_3,4_^2^, … *r_L_*_−1*,L*_^2^]. In the example shown in Table 1, with *L* = 10 loci and 45 locus pairs, any given pair shares one locus with 2 × 8 = 16 other pairs. The *r*^2^ values for pairs that share one locus (e.g., *r_i,j_*^2^ and *r_i,k_*^2^) will be positively correlated and hence will not provide independent information about LD.

In theory, pseudoreplication in LD could be quantitatively accounted for by specifying the relevant covariance matrix, but in practice, this is completely infeasible for genomics-scale datasets. The covariance matrix for *L* loci has order *L*^2^ elements, but this is not sufficient for quantifying this overlapping-pairs-of-loci problem, which requires one to specify correlations of correlations. The relevant covariance matrix therefore has order *L*^4^ elements, which rapidly becomes impossible to even contemplate for genomics-scale datasets.

In their study of pseudoreplication in large genomics datasets, Waples et al. [10] took a different approach, simulating many replicate datasets and measuring how rapidly the sampling variance in mean *r*^2^, Var(E[*r*^2^]), declined as it was averaged over more and more pairs of loci. If all the locus pairs were truly independent, the degrees of freedom associated with mean *r*^2^ would be *n* = *L*(*L* − 1)/2, and Var(E[*r*^2^]) would be inversely proportional to *n*. By quantifying Var(E[*r*^2^]) in their simulations, Waples et al. [10] were able to calculate an effective degree of freedom (*n*′) and compare it to *n* to quantify the magnitude of pseudoreplication. They found that for LD, the ratio *n*′/*n* increased with *N_e_* and the number of chromosomes and decreased as the number of loci increased; they also found that reductions in the *n*′/*n* ratio were primarily due to the overlapping-pairs-of-loci effect, with relatively little influence from physical linkage except when modeling species with relatively few numbers of chromosomes.

Here, we consider whether the concept of entropy can potentially be useful for evaluating pseudoreplication in studies of LD. Entropy metrics have previously been applied to genetic data [11,12], and to genetics problems involving functional information, but not to this specific problem—thus, this work adds to the growing body of literature comparing entropy-based approaches to other methodology. In contrast with functional information, which quantifies the information needed to achieve some threshold or function [13,14], our focus is on comparing two approaches for quantifying the information content of genomics datasets. First, we consider unlinked loci, which are easy to simulate on a desktop computer; this eliminates any lack of independence due to physical linkage, so any resulting pseudoreplication is due to overlapping pairs of loci. Second, we focus on measures of entropy to summarize the relationships among pairs of loci and variability in these relationships. Among the various entropy indices, we focus on total correlation (TC; [15]), which can be defined as the difference between two entropy measures:

(1) $$TC\left(x\right)=\sum H\left(x_{i}\right)-H\left(x_{1},x_{2},\;\ldots x_{n}\right)$$

where $H\left(x_{i}\right)$ is the information entropy of variable *x_i_* (locus pairs) and $H\left(x_{1},\;x_{2},\;\ldots x_{n}\right)$ is the joint entropy of the set [$x_{1},\;x_{2},\;\ldots x_{n}$]. TC(x) quantifies the amount of information that is shared within a dataset—hence, a lack of independence, or pseudoreplication. The first term in the above equation is the amount of information the variable set would contain if everything were independent, and the second term is the amount of information the variable set actually contains. We also consider entropy statistics summarizing H(x) as the total variability or spread of a dataset [16]. Based on results from [10], we predict that TC should be positively correlated with *L* and negatively correlated with *N_e_*.

## 2. Methods

### 2.1. Simulations

To investigate the utility of using entropy-based metrics to quantify the information about LD in genomics data, we created large simulated datasets from a monoecious population with random mating (including random selfing), using custom scripts in R 4.3.1 [17]. Each simulated dataset involved a different random, multi-generation pedigree; we initialized a random population consisting of *N_e_* parents, with each individual being heterozygous for each bi-allelic locus. We then projected these individuals forward for 6 generations by producing a constant number of *N_e_* offspring per generation, allowing individuals to reproduce randomly and alleles of offspring to be generated via Mendelian segregation. After the 6-generation burn-in period, which is sufficient to establish an equilibrium level of LD for unlinked loci [18], we then generated a sample of *S* = 50 or 100 offspring from the last generation of parents and removed monomorphic loci.

With single simulated datasets, one can calculate covariance matrices across loci, but those covariance matrices do not enable us to calculate the desired correlations of correlations. To calculate these higher-order correlations, we generated replicated datasets (*n* = 50) to provide an additional dimension, resulting in arrays of *r*^2^ (*L*, *L*, 50). With pedigrees or loci not being shared across replicates, there is the potential for pairwise correlations between loci to be eroded. To avoid these issues, each replicate involved sampling with replacement from the pool of potential offspring in the last generation. We next generated covariance matrices Σ and correlation matrices **R** across the *L*(*L* − 1)/2 pairs of loci.

To model infinite *N_e_*, we skipped the burn-in period, and, for each individual, drew genotypes at each locus randomly and independently, based on the parametric population allele frequencies. This generated single-locus genotypes in Hardy–Weinberg proportions, with random LD generated by sampling a finite number of offspring.

### 2.2. Quantifying Entropy

As a first entropy measure, we calculated Watanabe’s total correlation, TC(x), to summarize the interdependence among pairs of loci [15]. This measure is calculated as

(2) $$TC\left(x\right)=\sum ln\left(\lambda_{i}\right)-ln\left(\left|\Sigma \right|\right)$$

where the first term is the sum of logged eigenvalues from the covariance matrix, and the second term represents the log of the determinant (also calculated as the product of eigenvalues). In addition, we calculated entropy measures summarizing the variability in the covariance between pairs of loci. Assuming a multivariate normal distribution, this is calculated as

(3) $$H\left(x\right)=\frac{L}{2}\left(1+\ln(2\pi \right)+\frac{1}{2}ln\left(\left|\Sigma \right|\right)$$

Ref. [16], where Σ represents the covariance matrix estimated across the *L*(*L* − 1)/2 pairs of loci. This metric can be used to quantify the total information or spread of the *L*-dimensional space that a set of variables contains. Because covariance-based entropy is scale-dependent, we also calculated the same entropy measure on the correlation matrix **R**, as an invariant measure. While both TC(x) and H(x) are functions of the determinant of Σ or **R**, they represent different properties of the data—TC(x) quantifies dependency among loci, while H(x) is analogous to variation or uncertainty in a dataset [16].

There are several challenges in computing both entropy measures for large genetics datasets, as (1) the dimensionality of Σ increases with $L^{2}$, and (2) the correlations for many pairs of loci are very close to 0 (resulting in numerical instability). For each set of simulations, we used the RSpectra package [19] to calculate the first 500 eigenvalues, λ, corresponding to the eigen decomposition of Σ. We then calculated the log determinant as $\left|\hat{\Sigma }\right|=\overset{n_{p}}{\underset{i=1}{\sum }}\lambda_{i}$, where $n_{p}$ represents the number of positive eigenvalues. We used $\left|\hat{\Sigma }\right|$ to calculate H(x) and approximated

(4) $$TC=\left|\hat{\Sigma }\right|\left(1-\frac{n_{p}}{L}\right)$$

To understand the effects of changing population sizes or the dimension of genomics datasets, we conducted a sensitivity analysis across 2 orders of magnitude of values of *N_e_* (10–1240), an eightfold range of number of loci (*L* = 25–200), and a twofold range in size of the offspring population (*S* = 50–100; Table 2). Generating replicates or datasets was generally not computationally intensive for these sets of parameters; however, we did find computational challenges on desktop and laptop computers as the number of loci exceeded 200 (this directly affects the dimensionality of Σ). The suite of simulation parameters explored is given in Table 2.

### 2.3. Statistical Modeling

To evaluate the relative importance of the number of loci, *N_e_*, and the number of offspring on TC(x) and H(x), we analyzed the simulation output in a regression framework. Given the skewed distribution of TC(x) and H(x) in our simulated data, we used log (entropy) as the response variable in our regressions. Because H(x) is negative, we used log(−H(x)) as a response for models of H(x). We considered models using either raw or log-transformed predictor variables, and also evaluated models with linear interaction terms between loci, *N_e_*, and the number of offspring. Models were compared using AIC [20] and by examining the statistical significance of estimated coefficients. All regression modeling was performed using the R packages ‘stats’ (R Core Development Team 2023) and ‘glmmTMB’ [9]. Code and simulated data are provided in our Github repository for this paper, https://github.com/ericward-noaa/ward-waples-entropy (accessed on 1 August 2024).

## 3. Results

Because of the eigenvalue calculation, we found more computational challenges in calculating TC(x) than H(x) for large matrices (our simulation scenarios with 200 loci involved calculating entropy measures on matrices with 40,000 rows/columns and 1.6 × 10^9^ elements).

**Figure 1:**
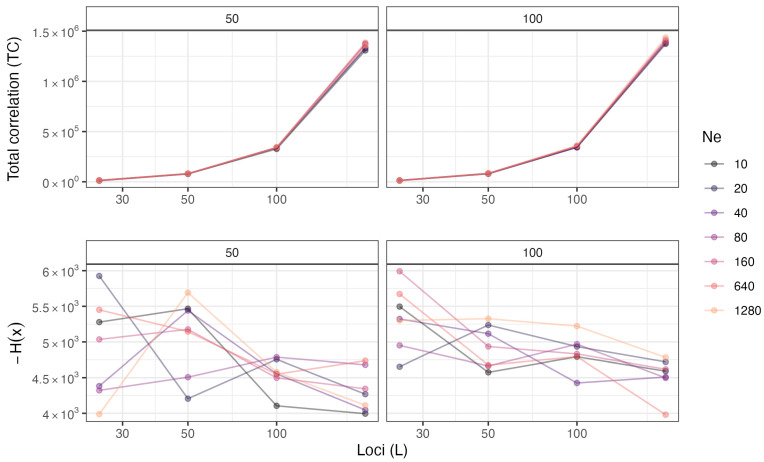
Relationship between entropy measures (TC and H(x), Y axes) in our simulation study, and the number of loci (*L*) (X axes; note the log scale). Results are presented for different values of *N_e_* (colors) and numbers of offspring (facets). If H(x) is modeled with a linear model with a common effect of loci (*L*) and factor levels for *N_e_*, the effect of loci is significant for both 50 (*p* < 0.01) and 100 offspring (*p* < 0.0001).

For linear models predicting log(TC) as a response, we found the most support for a model that did not include interactions between predictors; while we found positive associations with all covariates on *TC*, the effect (and statistical significance) was greatest for the effect of numbers of loci (Table 3, Figure 1). Though this model is relatively simple, it explains most of the variation in *TC* (R^2^ > 0.999). When fitting linear models to log(−H(x)), we again found the most support for not including interactions between variables. In contrast to models of *TC*, models predicting H(x) did not appear to explain much of the variation in the data (R^2^ < 0.12). Our simulated summary statistics of H(x) appeared more variable in general (Figure 1).

**Figure 2:**
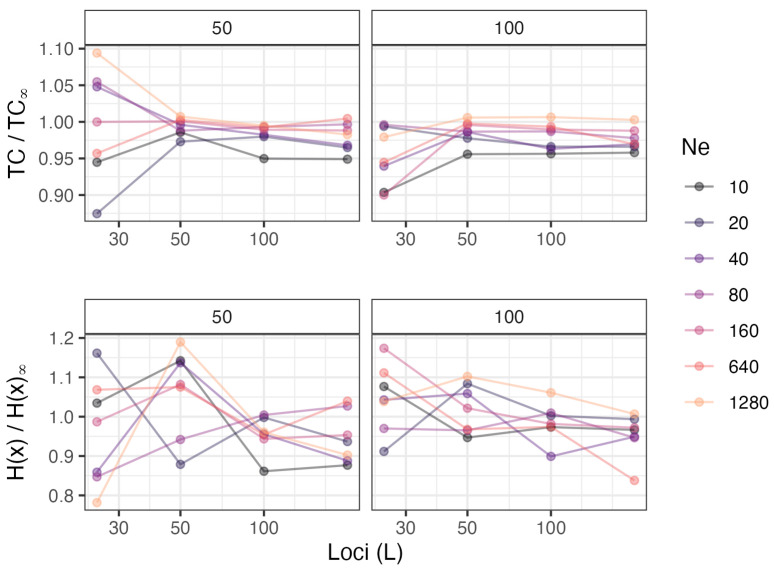
Relationship between scaled entropy measures (TC and H(x), Y axes) in our simulation study and the number of loci (*L*) (X axes; note the log scale). Results are presented for different values of *N_e_* (colors) and numbers of offspring (facets). On the Y axes, entropy measures for finite *N_e_* are scaled in comparison to results for infinite *N_e_*. If H(x) is modeled with a linear model with a common effect of loci (*L*) and factor levels for *N_e_*, the effect of loci is significant for 100 offspring (*p* < 0.008) but not for 50 offspring (*p* < 0.07).

Contrary to our hypothesis, we found little effect of *N_e_* on *TC* (Table 4). As a covariate, the effect of *N_e_* was not significant (Table 2), and influences of *N_e_* on *TC* are indistinguishable in Figure 1. The simulations modeling infinite *N_e_* were included to provide a reference point for evaluating the influence of effective population size. Waples et al. [10] found that when they modeled infinite *N_e_*, the observed Var(E[*r*^2^]) agreed closely with the expected variance, assuming all pairs of loci were completely independent—hence, pseudoreplication disappeared. We therefore hypothesized that as *N_e_* increased, *TC* should have converged on the value for infinite *N_e_*. Modest support for this hypothesis was found for results for 200 loci in the top-right panel in Figure 2: the ratio *TC*/*TC_∞_* was ~1.0 for the largest *N_e_* (1280), it was the lowest (~0.95) for the smallest *N_e_* (10), and results for the other modeled effective sizes were mostly in the hypothesized order.

**Table 2:** Parameters used in our simulation experiment; in addition to these parameters, we fixed the number of generations (*n* = 6) and replicates (*n* = 50).

Parameter	Values
*N_e_*	10, 20, 40, 80, 160, 640, 1280
Offspring (*S*)	50, 100
Loci (*L*)	25, 50, 100, 200

**Table 3:** Estimated predictors in linear models predicting total correlation, TC. The R^2^ from the model is >0.99. *L* is the number of loci and *S* is the number of offspring sampled.

Coefficient	Estimate	Std. Error	t value	Pr(>|t|)
Intercept	2.262085	0.205537	11.006	3.43 × 10^−15^
log(*L*)	2.211015	0.019244	114.891	<2 × 10^−16^
log(*N_e_*)	0.006825	0.009066	0.753	0.455
log(*S*)	0.050063	0.043032	1.163	0.25

**Table 4:** Correlation between total correlation, TC(x), the uncertainty represented by H(x), *N_e_*, the number of loci (*L*), and the number of offspring sampled (*S*).

	TC(x)	H(x)	*L*	*N_e_*	*S*
TC(x)	1.000	0.442	0.981	0.004	0.015
H(x)	0.442	1.000	0.514	−0.148	0.065
*L*	0.981	0.514	1.000	0.000	0.000
*N_e_*	0.004	−0.148	0.000	1.000	0.000
*S*	0.015	0.065	0.000	0.000	1.000

## 4. Discussion

The application of entropy-based summary statistics of pseudoreplication offers new insights into the nature of genetic data derived from genomics datasets. Entropy has the potential to serve as a more nuanced measure of the information contained within a dataset, capturing not just the amount but also the structure of genetic variation. Results from our simulation study confirm that total correlation (TC) increases with the number of loci, consistent with the results from [10]. This positive correlation underscores the challenges faced when attempting to interpret LD measures in the presence of extensive genomic data. As the number of loci increases, the assumption of independence among locus pairs becomes increasingly unrealistic, leading to inflated estimates of shared information, or pseudoreplication. This problem is compounded by the fact that entropy measures also become more challenging to compute as the dimensionality of the data increases, resulting in computational intractability for datasets at the higher end of the genomic scale.

Our study also highlights the nuanced impact of effective population size (*N_e_*) on the degree of pseudoreplication. The effects of *N_e_* in our simulations appear largest for smaller populations and small numbers of loci (*L*); however these combinations of parameter values also result in the most variability. For larger populations or scenarios with more than 100 loci, the effect of small samples diminishes and the effect of *N_e_* becomes much smaller. These results demonstrate a complex—and nonlinear—effect of *N_e_*, suggesting that the relationship may also be influenced by other factors such as the number of chromosomes.

In practical terms, our results suggest that when dealing with large genomic datasets, researchers need to be cautious in their interpretation of LD and related statistics. Traditional measures that assume independence among loci may be misleading, and the effective degrees of freedom associated with mean r^2^ may be substantially lower than the nominal number of locus pairs—entropy-based approaches represent one potential approach for better estimating the effective degrees of freedom. We considered two related entropy-based measures to quantify the effect of pseudo-replication. While entropy-based metrics such as TC and H(x) provide valuable tools for quantifying pseudoreplication, their utility may be limited in many real world applications by computational feasibility. Both metrics considered in our analysis rely on the eigen decomposition of very large sparse matrices; the spectral decomposition approaches used here may be applied to larger datasets; however the computational storage of large pairwise matrices (larger than 40,000 × 40,000) on desktop computers may become a greater limitation. Alternative solutions to increasing CPU or RAM include performing computations on high-performance computing clusters or utilizing graphics processing units (GPUs); such solutions were not explored in our analyses but may be useful for future extensions. Many other entropy-based metrics have also been advanced in fields with large datasets, such as machine learning [21], and similar approaches may be useful for genomics datasets.

The simulation assumptions used in our study were designed to be simplistic, but could be extended to other case studies. Examples include more realistic two-sex models, or case studies involving more complicated mating scenarios. Despite the increased realism, these advances will not solve the dimensionality constraints. A more promising and critical future area of research is developing efficient algorithms and computational techniques to calculate metrics such as TC on datasets with thousands or more loci. Leveraging computational innovations from fields such as data science and could bridge the current capability gaps, and also might lead to scalable entropy-based methodologies that could transform the landscape of genomic analysis, affecting diverse biological disciplines, from ecology to medicine.

## Figures and Tables

**Table 1:** The upper triangle of the matrix below shows the 10 × 9/2 = 45 different pairs of *L* = 10 gene loci. Considering the pair [2,9] (**•**), there are 16 other pairs (denoted by ‘X’) that share either locus 2 or locus 6, so *r*^2^ values for all these locus pairs are positively correlated. The other 28 locus pairs are not correlated with [2,9], but many are correlated with each other. Similar entanglements apply to every cell in this matrix.

Loci	1	2	3	4	5	6	7	8	9	10
1		O	X	O	O	X	O	O	O	O
2			X	X	X	•	X	X	X	X
3				O	O	X	O	O	O	O
4					O	X	O	O	O	O
5						X	X	X	X	X
6							O	O	O	O
7								O	O	O
8									O	O
9										O
10										

## Data Availability

Data are contained within the article.
